# Splicing of the rSlo Gene Affects the Molecular Composition and Drug Response of Ca^2+^-Activated K^+^ Channels in Skeletal Muscle

**DOI:** 10.1371/journal.pone.0040235

**Published:** 2012-07-10

**Authors:** Maria Maddalena Dinardo, Giulia Camerino, Antonietta Mele, Ramon Latorre, Diana Conte Camerino, Domenico Tricarico

**Affiliations:** 1 Departments of Pharmacobiology, Faculty of Pharmacy, University of Bari, Bari, Italy; 2 Centro Interdisciplinario de Neurociencias de Valparaíso, Facultad de Ciencias, Universidad de Valparaiso, Valparaiso, Chile; University of Queensland, Australia

## Abstract

The molecular composition and drug responses of calcium-activated K^+^ (BK) channels of skeletal muscle are unknown. Patch-clamp experiments combined with transcript scanning of the Kcnma1 gene encoding the alpha subunit of the BK channel were performed in rat slow-twitch soleus (Sol) and fast-twitch flexor digitorum brevis (FDB) skeletal muscles. Five splicing products of the Kcnma1 gene were isolated from Sol and FDB: the e17, e22, +29 aa, Slo27 and Slo0 variants. RT-PCR analysis demonstrated that the expression of e22 and Slo0 were 80–90% higher in FDB than Sol, whereas the expression of Slo27 was 60% higher in Sol than FDB, and the +29 aa variant was equally expressed in both muscle types. No beta 1-4 subunits were detected. In Sol, a large BK current with low Ca^2+^ sensitivity was recorded. The BK channel of Sol also showed a reduced response to BK channel openers, such as NS1619, acetazolamide and related drugs. In FDB, a reduced BK current with high Ca^2+^ sensitivity and an enhanced drug response was recorded. The total BK RNA content, which was 200% higher in Sol than in FDB, correlated with the BK currents in both muscles. Drug responses primarily correlated with e22 and Slo0 expression levels in FDB and to Slo27 expression in Sol muscle. In conclusion, phenotype-dependent BK channel biophysical and pharmacological properties correlated with the expression levels of the variants in muscles. These findings may be relevant to conditions affecting postural muscles, such as prolonged bed-rest, and to diseases affecting fast-twitch muscles, such as periodic paralysis. Down-regulation or up-regulation of the variants associated with pathological conditions may affect channel composition and drug responses.

## Introduction

Ca^2+^-activated K^+^ channels (BK), which are present in virtually every cell, couple chemical signaling to electrical signaling [1]–[2]. All BK channels are activated by increases in the concentration of intracellular Ca^2+^ ions, and many can be modulated by other messengers, such as protein kinases, phosphatases, and G proteins [3]–[8]. By damping excitatory stimuli mediated by the entry and/or the release of Ca^2+^ from internal stores, BK channels control diverse physiological processes, including the regulation of vascular tone [9]–[12], neuronal excitability [13]–[14], neurotransmitter release [15]–[16], endocrine function [17]–[19], innate immunity [20], and hearing [21]–[22].

BK channels in native tissues exhibit a physiologically diverse array of phenotypes. At least three major post-transcriptional mechanisms are involved in generating such functional diversity: the alternative pre-mRNA splicing of the BK channel pore-forming alpha-subunit; the assembly of alpha-subunits with a family of modulatory beta-subunits; and metabolic regulation (e.g., phosphorylation). A BK channel assembles as tetramers of the pore-forming alpha -subunit and is encoded by a single gene (Kcnma1) [23].

Electrophysiological recordings in native cells have revealed Slo1 channels with different calcium sensitivities. However, the Slo1 channel is encoded by a single gene in mammals. This channel diversity is possibly due to the alternative processing of introns, which produce at least 11 splice variants expressed in different tissues and cell types [24]. This feature is evolutionarily conserved and is observed in mammals, reptiles, birds and insects [23]–[31]. When expressed in heterologous expression systems, channels formed by these splice variants present different calcium sensitivities and gating kinetics, resembling those found in native cells. Alternative splicing is responsible in part for the great variety of calcium sensitivities among Slo1 channels. Several of these splice variants are produced by “insertions” at the C-terminus, and one of the most studied variants is expressed under the activation of the hypothalamic-pituitary-adrenal axis (HP) [32]–[33]. Two splice variants produce dominant-negative subunits, which retain the channel in subcellular compartments [34]–[35]. One of these variants corresponds to an insertion of 33 amino acids in S0 (SV1 subunit) to produce a natural dominant-negative subunit that reduces the expression level of Slo1 in the myometrium.

Analysis of individual alternatively spliced variants generated at distinct splice sites in different species has revealed that alternative pre-mRNA splicing can dramatically modify the functional properties of the BK channel alpha -subunit, including changes in calcium and voltage sensitivity [24]–[26], [36]–[39], regulation by protein phosphorylation [40]–[42], and other intracellular signaling cascades [43] as well as in cell surface expression [44]. Whether this post-transcriptional mechanism is operative in skeletal muscle and contributes to the formation of functional BK channels is not currently known.

The BK channel of the neuromuscular apparatus plays a role in coupling the intracellular calcium transient in the t-tubule system with the repolarization phase of action potentials, particularly during high-frequency firing. Slow-twitching and fast-twitching skeletal muscles serve different functions, such as postural maintenance and voluntary contraction. The two muscle phenotypes can be distinguished by their contractile proteins, cellular metabolism, hormonal regulation/drug responses, and sarcolemmal ion channel activity [45]. Patch-clamp investigations have identified a BK channel population in slow-twitch muscles that exhibits functional properties different from those of BK channels present in fast-twitch muscles [45]. Whether the functional diversity of the BK channel subtypes observed in the different skeletal muscle phenotypes correlates with alternative splicing of the Slo gene or with the assembly of the accessory beta 1–4 subunits is currently unknown.

The BK channel is also a molecular target for drugs of therapeutic interest for periodic paralysis, such as acetazolamide and dichlorphenamide. Periodic paralysis is a group of neuromuscular disorders characterized by fiber depolarization and flaccid paralysis of fast-twitch muscles associated with changes in serum K^+^ ions [46]. In these disorders, acetazolamide and dichlorphenamide act by opening skeletal muscle BK channels [46]. The reasons why some patients do not respond to drug treatment or show detrimental responses are unclear [46]–[47]. Despite the important roles of BK channels in skeletal muscle functions, the molecular composition and the drug responsiveness of the different BK channel subtypes in slow-twitch and fast-twitch muscles are currently unknown.

In the present work, transcript scanning of the Kcnma1 gene was performed in combination with patch-clamp experiments on rat slow-twitch soleus (Sol) and fast-twitch flexor digitorum brevis (FDB) skeletal muscles to investigate the molecular composition, biophysical properties and drug responses of BK channels. RT-PCR experiments were performed for the quantitative evaluation of the identified splicing products in the two muscle phenotypes.

We have determined that the assembly of different splicing products of the alpha subunit gene leads to different BK channel phenotypes in skeletal muscles. The muscle phenotype-dependent properties and drug responses of BK channels depend on the distribution of the observed isoforms. These findings may have relevance for conditions affecting mainly postural muscles, such as prolonged bed-rest and microgravity, or conditions affecting fast-twitch muscles, such as periodic paralysis. The down-regulation or up-regulation of these variants associated with pathological conditions may alter channel composition and drug responses.

## Results

### Analysis of Variants of Rat Kcnma1 at the N1 and C1-6 Splice Sites

**Figure 1:**
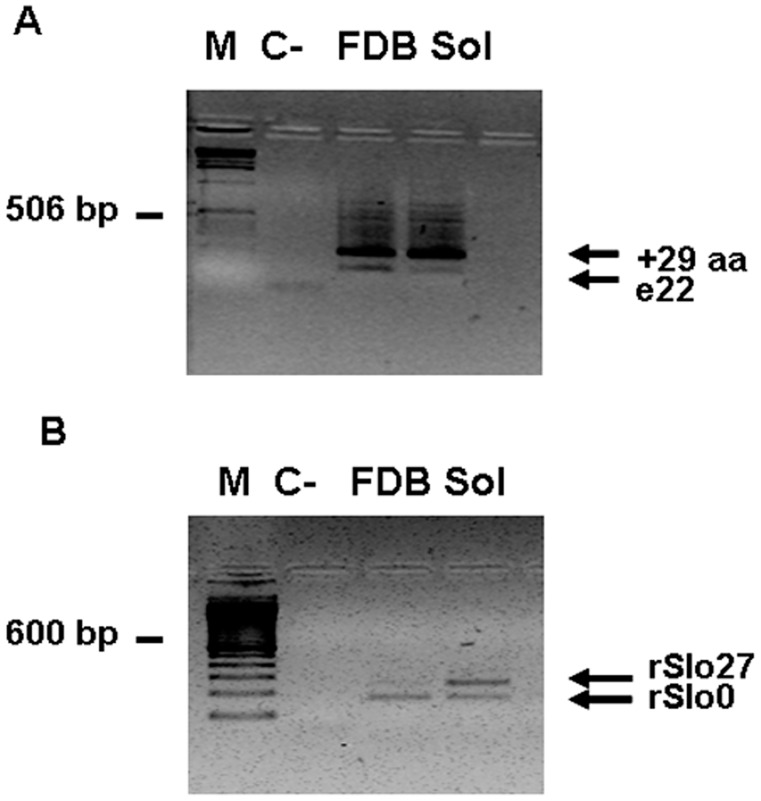
Nested PCR of BK channels at sites of alternative splicing C2 and C4. (A) Representative ethidium bromide-stained agarose gel of amplicons generated from a PCR screen of rat FDB and Sol cDNA in the region of the alternative splice site C2. The gel shows two bands of different molecular weights for rat FDB and Sol, corresponding to the isoforms e22 and +29 aa. M, marker; C- negative control. (B) Rat BK PCR at alternative splice site C4. Representative ethidium bromide-stained agarose gel of amplicons generated from a PCR screen of rat FDB and Sol cDNA in the region of alternative splice site C4. Amplicons are representative of PCR screens of two (a and b) primers of Sol (S) and FDB (F). Two splicing isoforms are shown with the “a” primers. M, marker; C- negative control.

Numerous splice sites have been reported for the Kcnma1 gene in humans and mice [33], [35]. In the present study, we searched for the presence of splicing isoforms of BK channels in skeletal muscle by analyzing the regions that are known to contain splice sites in most BK channels. Transcript scanning of cDNA generated from RNA from rat Sol and FDB skeletal muscles was performed using primers designed to amplify across the alternative splice sites C1 and C2, between exon 15 and exon 25. PCR amplification revealed two alternatively spliced transcripts in Sol and FDB samples, with distinct inserts at the alternative splice sites C1 and C2 (Table 1). At site C1, we isolated one variant that lacked exon 17; this exclusion eliminates the 4 amino acids RSRK from the protein in both muscle types. At site C2, two distinct variants were isolated: the e22 variant, resulting from the inclusion of exon 21 between exon 19 and exon 22, and the +29 variant, in which part of intron 22 was added to the sequence of the e22 variant. To address the distribution of each C2 site splice variant mRNA in Sol and FDB rat skeletal muscle, we performed nested PCR using internal primers targeting the region between exon 19 and exon 22 (Table 2). The product of this amplification was present in both muscles, but was expressed more highly in the fast-twitch muscle FDB (Fig. 1A).

**Table 1:** Splicing sites and relative amino acid sequences of rat Soleus (Sol) and Flexor digitorum brevis (FDB) muscles Kcnma1 gene.

Splicing site	Isoforms	Sequence
N1	No splicing	
C1	e17	
C2	e22+29 aa (hbr5)	KVEARARYHKDPFMHKNATPNSPHVPKPVSRLKLEHAITKTHLCTRMQLPILNTCPSQ
C3	No splicing	
C4	rSlo0rSlo27	AKPGKLPLVSVNQEKNSGTHILMITEL
C5	No splicing	
C6	No splicing	

**Table 2:** Primers used for PCR reactions.

Primer name	Nucleotide sequence (5′- 3′)	Annealing temperature (Tm)
**BKC2for**	GTCCTTCCC TAC TGT TTG	54°C
**BKC2rev**	GTG TTT GAG CTCATG ATA GT	56°C
**BKC1for**	TGACGTCACAGATCCCAAAA	58°C
**BKC1rev**	GGATGTGTTGGGTGAGTTCC	66°C
**BKC2rev2**	AGCGTGCTCTAGCTTCAACC	66°C
**BKC4for**	ACACCTCCTGGAATGGACAG	62°C
**BKC4rev**	TGATGACCCTGACACAGAGC	62°C
**BKC4rev2**	CAGTGGGACGCACATTCTAA	60°C
**BKC3for**	CCGAAGCTGATGAGGCATGA	58°C
**BKC3rev**	GAATCCTTGGGAATTAGCCT	58°C
**BKN1for**	AGGAGGTGGTGGCAGCCGA	64°C
**BKN1rev**	AAACCGCAAGCCAAAGTAGAG	62°C
**BKC6for**	TGTACCTCACACAGCCCTTT	60°C
**BKC6rev**	TCATCTGTAAACCATTTCTTTT	56°C
**Ratbeta1for**	CCCCAGGAACCTGGACAACTA	66°C
**Ratbeta1rev**	GCTCAACAGGTCCCTCTCTG	64°C
**Ratbeta2for**	AGGAGTCCATGTCCCTTGTG	62°C
**Ratbeta2rev**	GAGAGGATCCAACGGATCAA	60°C
**BKbeta3F**	CTCGGGACAGAAAGTGCTTC	62°C
**BKbeta3R**	GACAACCTTGTGCCAGACT	62°C
**BKbeta4F**	TCCTGACTAACCCCAAGTGC	62°C
**BKbeta4R**	AAGCAGTGCAGGAGGACAAT	60°C

No differences in the expression of Kcnma1 were found in Sol and FDB muscles for the +29 variant. Fig. 1A shows that this variant resulted in bands of the same length and intensity for the two types of muscle. C4 is an important splice site because it is located near the Ca^2+^-bowl, between segments S9 and S10 [38]. The resulting splice variant has a 27-amino acid insert that is most likely involved in the modulation of Ca^2+^ signaling. PCR amplicons of this site were cloned, characterized and sequenced (Fig. 1B). We isolated two products for Sol and FDB, represented by the +27 variant and the insertless variant. The +27 variant is expressed more highly in Sol, as confirmed by a second round of PCR performed with a reverse primer that anneals to the sequence of the 27-amino acid insert (Fig. 1B).

In a second step, we analyzed the variants of rat Kcnma1 at other hypothetical splice sites reported in the literature that had not been functionally characterized. In particular, we concentrated on the sites C3, C5 and C6 for the C-terminal region and on the N1 site for the N-terminal region of the Kcnma1 cDNA. Therefore, we amplified, cloned and sequenced the fragments containing these sites, and the results were aligned with the rat Kcnma1 CDS from the NCBI database (NM_031828). For the N1, C5 and C6 sites, we found no splicing of the analyzed regions, more or less equally distributed in Sol and FDB. PCR amplification of the C3 site region revealed no splicing in Sol and was not amplified in FDB, perhaps because of a polymorphism in a primer-binding site or the reduced expression of the C3 site. Therefore, we investigated the C3 site in FDB using the same primers but increasing the number of PCR cycles (Table 2). Using this approach, we found that the C3 site was amplified in FDB muscle, indicating that there was no difference in the cDNA sequence at this site in Sol and FDB muscles and that the C3 site was expressed at a lower level in FDB.

### Quantitation of Kcnma1 Splice Variants in FDB and Sol Muscles Using TaqMan qRT-PCR

To determine the isoform distribution of BK channels in slow- and fast-twitch skeletal muscles, we measured mRNA levels in Sol and FDB using real-time PCR assays with TaqMan MGB probes that could distinguish between splice variants. In particular, we focused on the three most informative splice sites: C1, C2 and C4.

**Figure 2:**
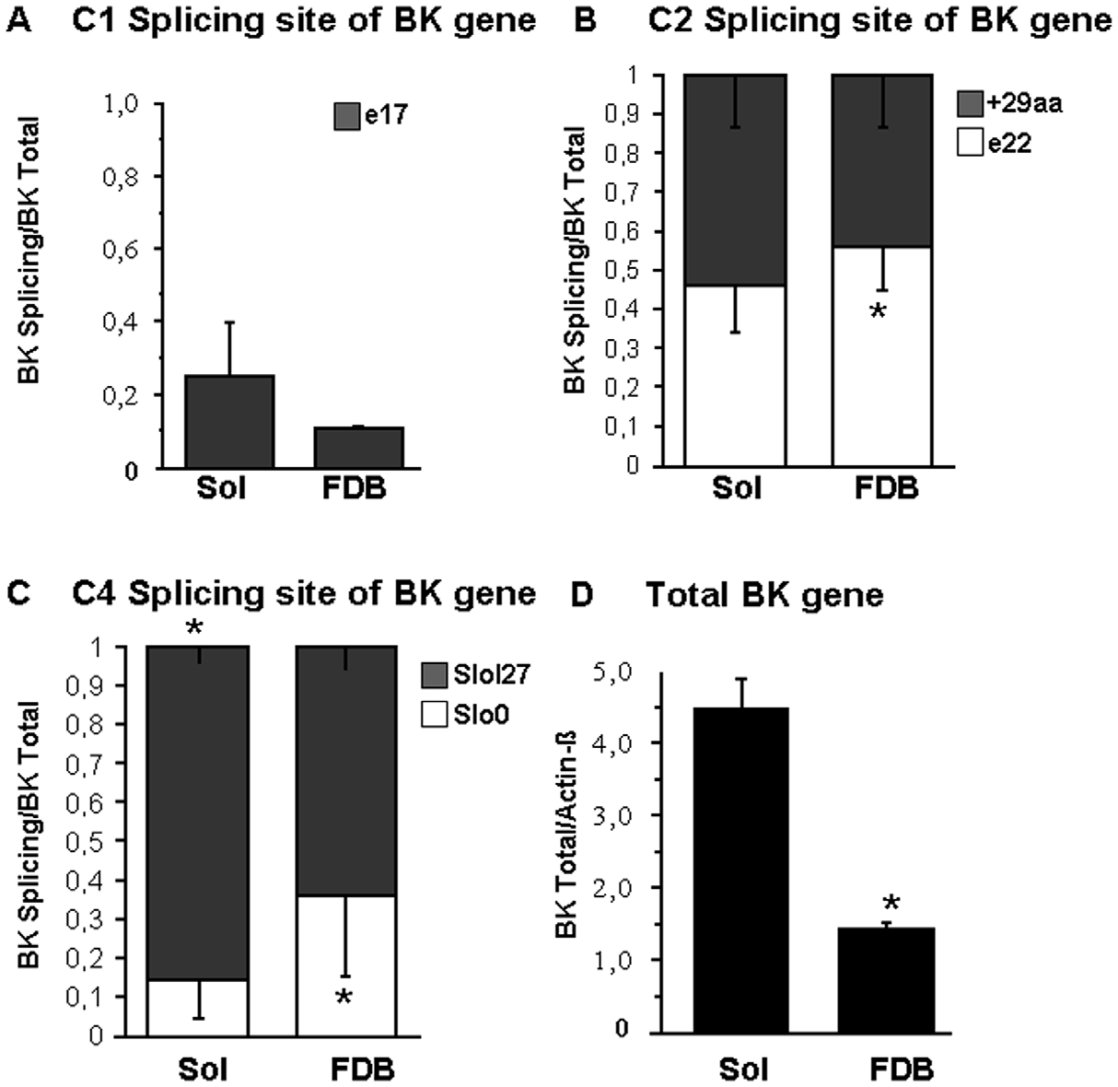
Differential tissue distribution of BK channel splice variant mRNA. mRNA levels of C1 (A), C2 (B) and C4 (C) splice variants in Sol and FDB expressed as a fraction of total BK channel mRNA expression in each tissue. (D) Differential tissue distribution of total BK channels. mRNA expression of total BK channels is normalized to the expression of beta -actin. Significant differences between data are evaluated by the unpaired Student’s t-test at p<0.05.

We designed two probes for the C1 region: one that anneals to the nucleotide sequence that encodes the RSRK motif of the protein and another that anneals outside of this region (Table 3). The first probe characterizes the e17 variant, and the second probe characterizes the variant with the 4-amino acid insertion. The lack of amplification by the real-time PCR assay for the region with the 4-amino acid insertion confirms the absence of exon 17 in all samples tested (Fig. 2A).

**Table 3:** Primers and probes used for TaqMan™ quantitative real-time PCR (qRT-PCR) assays.

Gene/Splicing	PROBE MGBNFQ 6-FAM	PRIMER FOR	PRIMER REV
**BK full lent**	CAGAGTCCTGGTTGTGTTA	GCTGGATGACATCTGTGAAGG	GCACCAATGCTGAGAGCAAA
**β-Actin**	TGAAGATCAAGATCATTGCT	GAGATTACTGCCCTGGCTCCTA	CTGCTTGCTGATCCACATCTG
**BK C2 e22**	CTCATCTTCAAGCCGCC	CCTCATGCCCCCATTACGT	CAGATCCCAAAAGAATTAAAAAATGC
**BK C2 87+**	CTAGAGCACGCTATCAC	GCAGGCGGCTCAAGGTT	GCGGTTGCTCATCTTCAACTG
**BK C4 Slo27**	ACGGAACTCGCTAAGC	CGGAGTCAACATTCCCATCAT	TTGACTGATACCAAAGGCAAC
**BK C4 Slo0**	CACGGAACTCGTGAAC	TCCATCACAACCGGAGTCAA	GTACAGCTCTGTGTCAGGGTCATC
**BK C117 null**	AACAGGGAGAGCCGAATA	GCTCCTGATGATAGCCATTGAGT	AAGGTGGTTCCCAGGGTTAATT
**BK C117 full**	TGGGAACCACCTTAAG	CGAAGCCGAAAGCGAATATT	TTGCGATGAAAAATCCTAAAGTACCT

We designed two probes targeting the C2 site: one specific for the e22 isoform and another specific for a 29-amino acid insertion, which we found and characterized by cloning and sequencing. Real-time PCR experiments showed that the +29 isoform was equally expressed in FDB and Sol muscles; e22 was expressed more highly in FDB (Fig. 2B).

We designed two probes against the C4 site that could distinguish the Slo27 variant with an insertion of 27 amino acids from the insertless Slo0 variant. The Slo27 variant was expressed more highly in Sol than in FDB, whereas Slo0 was more abundant in FBD than in Sol (Fig. 2C).

Real-time PCR amplification of a region of the gene that is not subject to alternative splicing demonstrated that the expression of the total BK channel was more abundant in Sol than in FDB, which is consistent with the recorded channel activity in both muscle types (Fig. 2D).

### Absence of BK Channel Beta-subunits in Slow-twitch and Fast-twitch Skeletal Muscle

**Figure 3:**
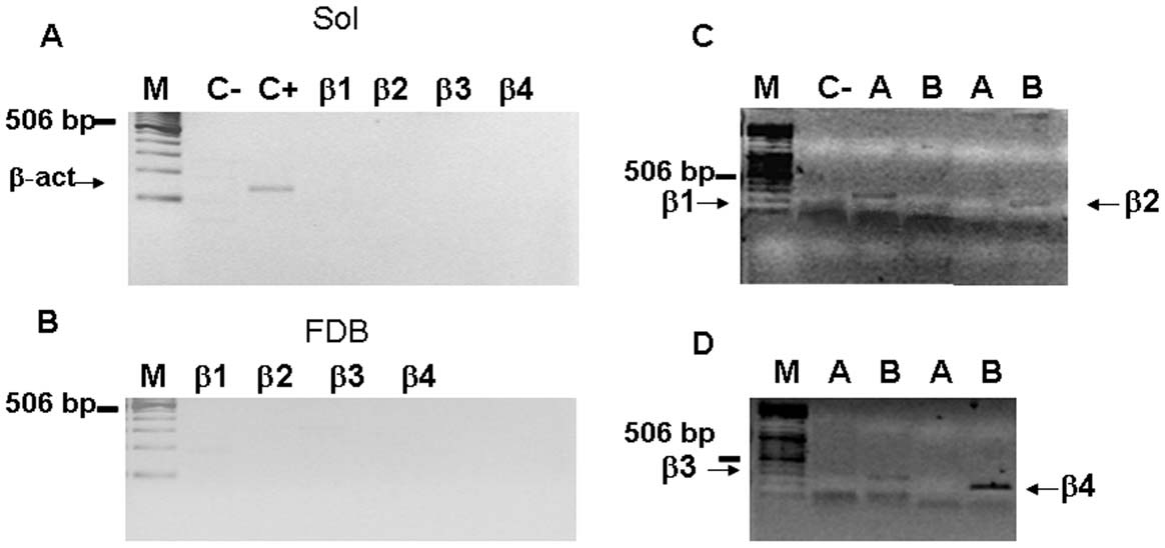
PCR of Rat BK accessory beta -subunits in different tissues. ( A–B) Representative ethidium bromide-stained agarose gel of amplicons generated from a PCR screen of rat Sol (A) and FDB (B) cDNA beta 1-4 subunits. M, marker; C- negative control; C+ positive control (rat actin). (C–D) Representative ethidium bromide-stained agarose gel of beta 1-4 subunits amplified by PCR on positive samples: brain B for beta 2-4 and aorta A for beta 1.

The properties of BK channels are influenced by accessory beta -subunits. We analyzed the cDNA of Sol and FDB to determine the presence of any of the four BK channel beta -subunits in rat skeletal muscles. The primers used in PCR reactions are reported in Table 2. None of the genes observed were amplified by this method, which likely disaffirms their expression in the FDB and Sol samples analyzed (Fig. 3A, B). This result was confirmed by testing these primers in tissues that normally express the beta -subunits under investigation, such as the brain and aorta from rats. PCR analysis showed that the brain expressed all subunits except beta-1 and that the aorta selectively expressed the beta-1 subunit (Fig. 3C, D).

### Muscle Phenotype-dependent Calcium Sensitivity and Drug Responses of BK Channels

**Figure 4:**
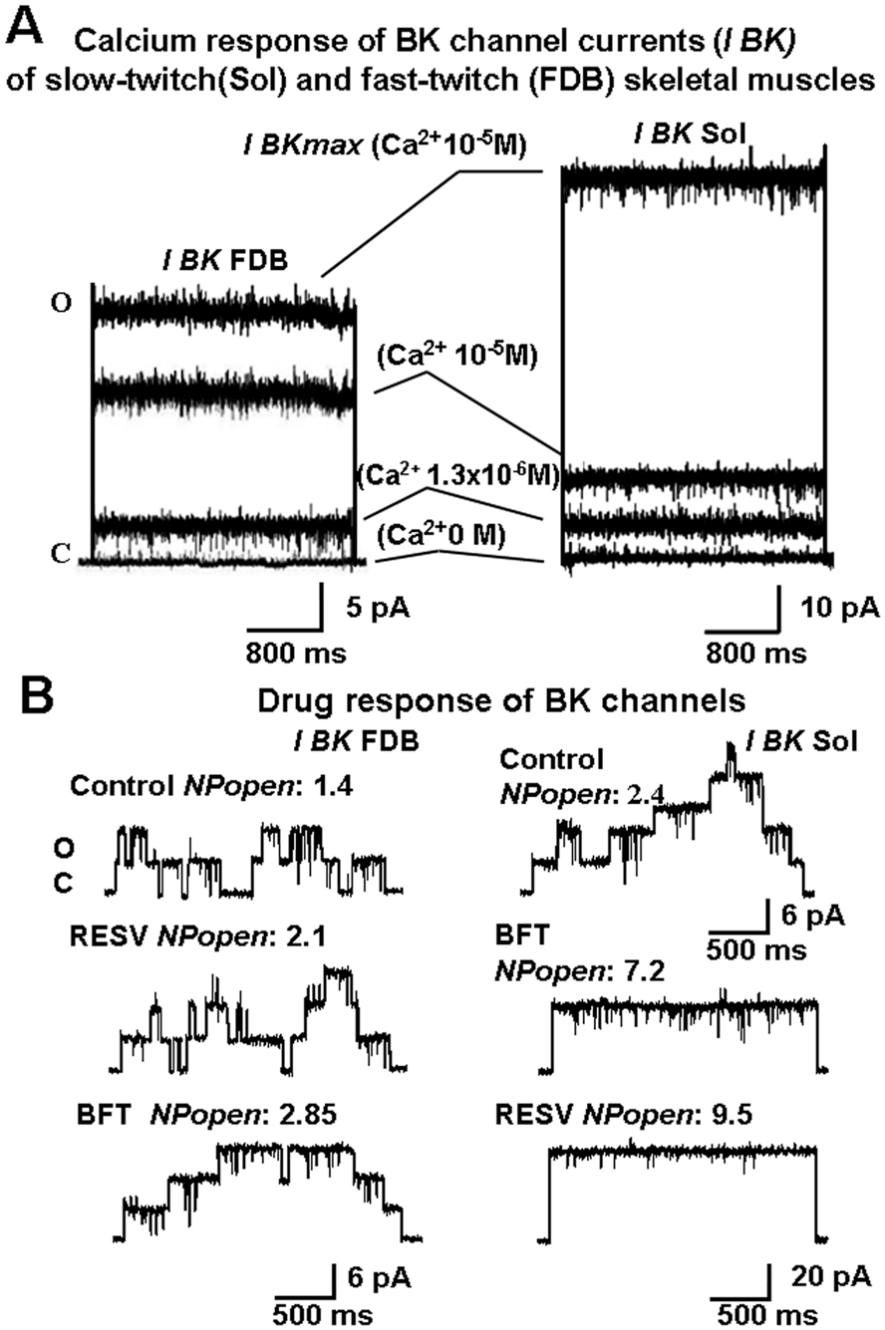
Calcium response and drug response of Ca^2+^-activated K^+^ channels (BK) of flexor digitorum brevis (FDB) and soleus (Sol) fibers. (A–B) Currents were recorded during voltage steps from 0 mV holding potential, prepulse of −70 mV, to −80 mV and +80 mV (Vm) in excised macropatches with 150 mM KCl on both sides of the membrane patches and in the presence of increasing calcium concentrations in the bath. Upward deflection in the current record indicates outward currents recorded at +30 mV or +80 mV (Vm); downward deflection indicates inward currents recorded at −60 mV (Vm). C, closed channel level; O, open channel level. The currents were leak-subtracted. (A) The represented BK channel current traces of FDB and Sol fibers were obtained by an ensemble average of 16 and 18 sweeps (1 sweep/voltage membrane/patch) from 16 and 18 patches for each FDB and Sol muscle, respectively. Channel currents were recorded in the FDB and Sol fibers using pipettes of 1.9±0.6 MOhm and 2.1±0.4 MOhm resistance, respectively. The Ca^2+^ response of the channels was evaluated at +30 mV (Vm) in the presence of increasing concentration of internal Ca^2+^ ions from 0 M to 1.3x10^−6^M and 10^−5^M. The recorded currents were than normalized to *I BKmax* which is the maximal BK current recorded at +80 mV (Vm) in the presence of 10^−5^M concentration of free Ca^2+^ ions. A maximal BK channel activation of 80% was observed in the FDB fibers in these patches in the presence of 10^−5^ M concentration of Ca^2+^ ions, whereas no more than 15% activation of the BK channel current was observed in Sol fibers in the presence of 1.3x10^−6^M and 10^−5^ M concentrations of Ca^2+^ ions indicating that the BK channel population in FDB fibers was more sensitive to Ca^2+^ ions than those of Sol fibers. (B) BK channel current response to resveratrol (RESV) (2x10^−4^M) and bendroflumethiazide (BFT) (2x10^−4^M) recorded in fibers from FDB and Sol muscles. Single channel currents were recorded in the FDB and Sol fibers using pipettes of 3.2 MOhm and 3.4 MOhm resistance, respectively. Single channel transitions were observed in the FDB and Sol fibers at +30 mV (Vm) in the presence of 10^−5^ M free Ca^2+^ ions. The *N Popen* was higher in the Sol fiber as compared with that recorded in the FDB fiber, the number of functional channels in the patches were 3 in the FDB fiber and 5 in the Sol fiber. No difference was observed in the single channel current between the FDB and Sol fibers. The application of RESV and BFT increased the *N Popen* of the channel without affecting single channel current. In the FDB fiber, BFT was more effective as BK opener than RESV, whereas RESV was more effective than BFT in the Sol fiber.

In FDB and Sol fibers, patch depolarization and the internal application of micromolar concentrations of free Ca^2+^ ions to the excised patches led to the activation of BK channels. Here, we showed that Sol fibers have higher BK channel activity in the physiological range of voltages than those recorded in FDB (Fig. 4A). In FDB fibers, BK channel currents recorded at −60 mV (Vm) in the presence of 1.3×10^−6^ M and 10^−5^ M concentrations of free Ca^2+^ were –0.911±0.02 pA/patch area and −4.51±0.041 pA/patch area (n = 100), respectively, and were 3.31±0.02/patch area and 16.2±002 pA/patch area (n = 100), respectively, at +30 mV (Vm) (Fig. 4A). These currents were sustained by 2–3 active channels per patch area. The exposure of the patches to increasing concentrations of free Ca^2+^ ions from 0 to 1.3×10^−6^ M enhanced the channel current to 9±1% and 21.4±3% at −60 mV and 30 mV (Vm), respectively. The increase in the Ca^2+^ ion concentration from 1.3×10^−6^ M to 10^−5^ M led to an enhancement of the channel currents to 21.4±2% and 87.7±4% at −60 mV and 30 mV (Vm), respectively. This result was observed in 85% of the patches examined, whereas the residual patches contained channels with lower sensitivity to Ca^2+^ ions.

In Sol fibers, BK channel currents recorded at −60 mV in the presence of 1.3×10^−6 ^M and 10^−5 ^M Ca^2+^ ions were −6.42±0.2 pA/patch area and −9.41±0.1 pA/patch area (n = 112), respectively, and 12.1±0.5 pA/patch area and 19.8±2 pA/patch area (n = 112) at +30 mV (Vm), respectively (Fig. 4A). These currents were sustained by 6–7 active channels per patch area. The exposure of the patches to increasing concentrations of free Ca^2+^ ions from 0 to 1.3×10^−6^ M enhanced channel currents to 7.6±2% and 14.64±4% at −60 mV and 30 mV (Vm), respectively. The increase in free Ca^2+^ ions from 1.3x10^−6^ M to 10^−5^ M led to an enhancement of the channel currents to 4.07±2% and 11.7±4% at −60 mV and 30 mV (Vm), respectively. This result was observed in 79% of the patches examined, indicating that most of the Sol BK channels have a lower sensitivity to Ca^2+^ ions than those of FDB fibers (Fig. 4A).

**Figure 5:**
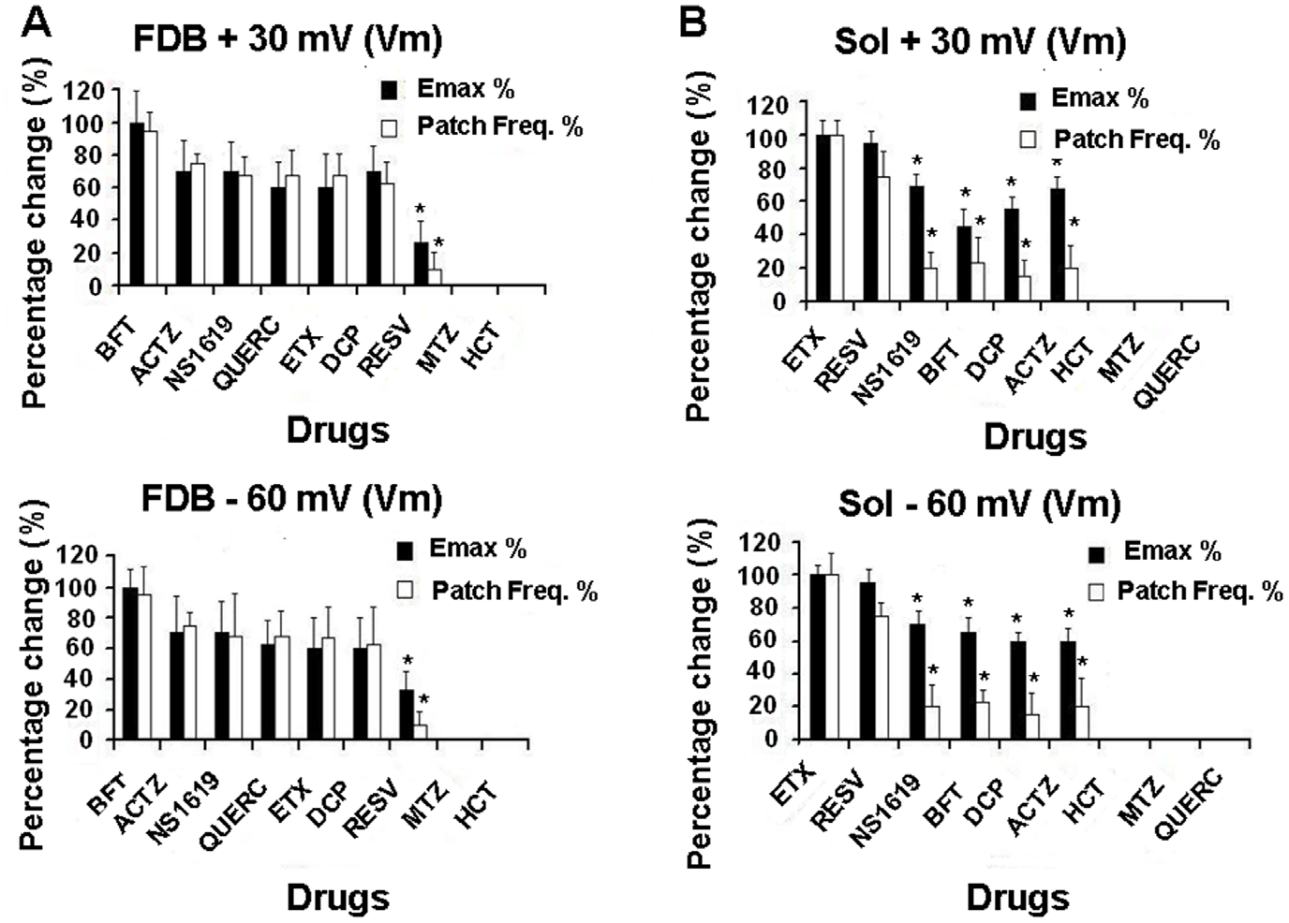
Frequency distribution (Patch freq.%) of patches containing Ca^2+^activated K^+^(BK) channels drug sensitive and the maximal drug effect (Emax%) at different voltages. Drug response was evaluated at 2x10^−4^M concentration in excised patch experiments at −60 mV and +30 mV (Vm) with 150 mM KCl on both side of the membrane patches, and in the presence of 10^−5^M Ca^2+^ ions concentrations in the bath. (A) An higher frequency of finding patches containing BK channels responders to bendroflumethiazide (BFT), acetazolamide (ACTZ), benzimidazolone (NS1619), quercetin (QUERC), ethoxzolamide (ETX), dichlorphenamide (DCP) was found in flexor digitorum brevis (FDB) fibers with respect to soleus (Sol) fibers at both voltages. A low frequency was calculated for resveratrol (RESV) in the same muscle fibers, while methazolamide (MTZ) and hydrochlorthiazide (HCT) were not effective. The data were expressed as means ±E.S. Data (*) were significantly different for P<0.05 in comparison to BFT data. (B) An higher frequency of finding patches containing BK channels responders to ETX and RESV was found in Sol fibers as compared with FDB fibers, but a low frequency of finding BK channels responders to ACTZ, NS1619, ETX, and DCP was found in these fibers. HCT, MTZ and QUERC were not effective in Sol fibers. Data (*) were significantly different for P<0.05 in comparison to ETX and RESV data.

In FDB fibers, BK channel openers were tested at a concentration of 2×10^−4^ M; with the exception of HCT and MTZ, which were not effective, all of the compounds activated BK channels, albeit with different degrees of effectiveness. BFT was the most effective drug in FDB fibers, inducing a 100% activation of the channel currents in 85% of the patches (Fig. 4B, 5A).

The effects of BFT on FDB fibers were not significantly different from those of DCP, ACTZ, NS1619, ETX and QUERC in the same patches. The BK channels activated by these drugs were also highly sensitive to Ca^2+^ ions. RESV was the least effective drug in FDB fibers, activating the low Ca^2+^-sensitive BK channels in 15% of the patches (Fig. 4B, 5A).

In Sol fibers, RESV caused a 95% activation of the channel current, whereas BFT produced a 50% activation of the channel current at +30 mV (Vm) (Fig. 4B). ETX and RESV were the most effective drugs in activating BK channels, causing a significant channel activation compared with that observed with NS1619, BFT, DCP and ACTZ which were effective in 15–20% of the patches (Fig. 5B). QUERC, MTZ and HCT failed to activate the BK channels of Sol fibers.

**Figure 6:**
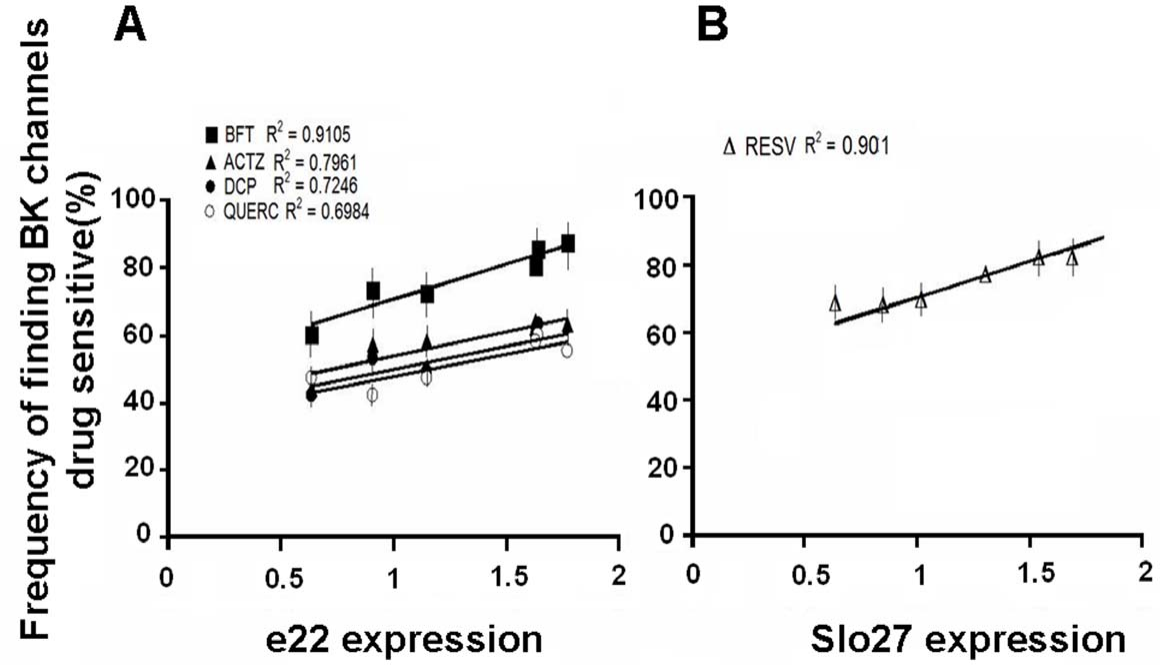
Correlation between the e22 and Slo27 expression levels and drug responses in different muscle phenotypes. The linear regression analysis between the e22 and Slo27 expression levels and the frequency distribution of patches containing Ca^2+^activated K^+^(BK) channels sensitive to bendroflumethiazide (BFT), acetazolamide (ACTZ), dichlorphenamide (DCP), and quercetin (QUERC) in flexor digitorum brevis (FDB) (A) or resveratrol (RESV) in soleus (Sol) (B) fibers for each muscle sample was performed. The e22 and Slo27 expression levels were normalized to the total BK. The correlation coefficient (R^2^) is reported for each drug.

A correlation analysis between the percentage of patches containing BK channels that were sensitive to BFT and the expression of e22 normalized to the full-length transcript in each FDB muscle sample showed a linear relationship, with a calculated correlation coefficient of 0.91. Correlation coefficients of 0.79, 0.72 and 0.69 were calculated for ACTZ, DCP and QUERC, respectively (Fig. 6A). Correlation coefficients of 0.66 and 0.65 were calculated for NS1619 and ETX, respectively. However, a lower correlation coefficient (0.62–0.59) was calculated between the rSlo0 expression level and the response to drugs. No correlation was found between the percentage of patches containing BK channels sensitive to the drugs and the expression level of the Slo27 variant in Sol muscle, with the exception of RESV, which showed a correlation coefficient of 0.9 (Fig. 6B). ETX showed a lower correlation coefficient of 0.65. No significant correlation was found between the expression levels of the +29 aa variant in FDB and Sol muscles and drug responses.

## Discussion

### Identification of Alpha-subunit Variants in Slow-twitch and Fast-twitch Skeletal Muscle and Absence of Beta Subunits

Analysis of variants of the rat KCNMA1 gene at splice sites N1 and C1-6 revealed the presence of 5 different known variants in both FDB and Sol muscles, such as e17 in C1, e22 and +29 aa in C2 and rSlo27 and rSlo0 in C4, and differences in the distribution of the isoforms were found between muscle phenotypes. Indeed, quantitative RT-PCR experiments showed that e22 and rSlo0 were expressed more highly in fast-twitch than in slow-twitch muscles. However, Slo27 was expressed more highly in slow-twitch than in fast-twitch muscles. The e22 splicing isoform is known to be responsible for increased Ca^2+^ and voltage sensitivity and regulation of BK channels by PKA phosphorylation in cell lines, which may account for the observed differences in Ca^2+^ sensitivity between the muscle phenotypes observed in our experiments [33]. The enhanced Ca^2+^ sensitivity of the BK channel in the fast-twitch muscle can be helpful for coupling fast Ca^2+^ transients, which are characteristic of this muscle phenotype, with the repolarization phase of the action potential, particularly during firing.

The BK channel alpha-subunit was more abundant in slow-twitch than in fast-twitch muscle, which is consistent with the elevated recorded channel currents observed in slow-twitch muscle. It is likely that the Slo27 variant is responsible for the high expression level of BK channels in this muscle phenotype.

Because the properties of BK channels are influenced by the accessory beta-subunits (beta1-4) [48]–[50], we analyzed the cDNA of the four beta-subunits of BK channels in Sol and FDB to determine their presence in rat skeletal muscles. None of the genes observed were amplified by this method, likely excluding their expression in the samples analyzed. In contrast with other tissues in which the beta 1–4 subunits play a significant role in determining the properties of BK channels, the differences in the BK channel populations in the fast-twitch and slow-twitch muscles in our experiments are due to the differential regulation of the expression of each splicing isoform.

### Pharmacological Response of BK Channels in Fast-twitch and Slow-twitch Skeletal Muscles

Analysis of drug responses by BK channels showed that BFT, ACTZ, DCP, NS1619 and QUERC were more effective on fast-twitch muscle BK channel subtypes than on slow-twitch muscle BK channel subtypes. The drug actions correlated with the expression level of the e22 and rSlo0 variants, suggesting that these isoforms can be the molecular targets for these drugs. In contrast, RESV was a weak agonist of the fast-twitch BK channel but was the most effective BK channel opener in the slow-twitch fibers. The action of RESV correlated with the expression level of the Slo27 variant in this tissue, suggesting that it may be the molecular target for this drug. The observed action of RESV as a skeletal muscle BK channel opener may help to explain the emerging use of this drug in the treatment of atrophy in human and animals [51].

**Figure 7:**
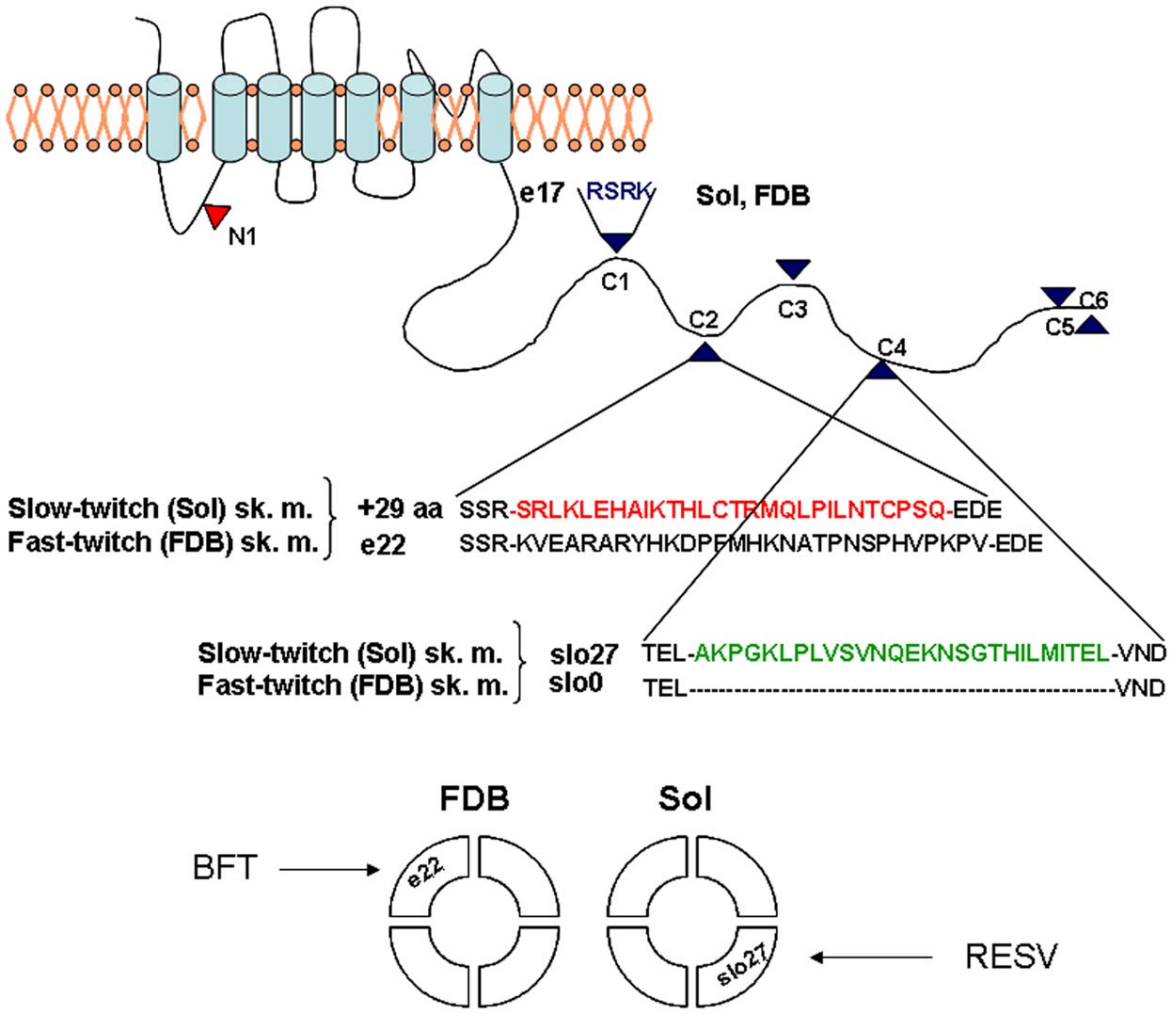
Schematic illustrating BK channel structure and sites of variable splicing in the BK alpha-subunit of slow-twitch soleus (Sol) and fast-twitch flexor digitorum brevis (FDB) skeletal muscles, and the related channel models. Five variants were found in the slow-twitch and fast-twitch skeletal muscles: e17 in C1, +29aa or e22 in C2, and Slo27 or Slo0 in C4 sites. These variants participate in the composition of the tetramers. The variants e22 and Slo27 characterizing the BK channels of the FDB and Sol muscle phenotypes, respectively, are shown. BK channels containing e22 variant are sensitive to bendroflumethiazide (BFT) while those containing Slo27 are sensitive to resveratrol (RESV).

Several forms of BK channels can be functionally active in fast-twitch and slow-twitch muscles, differing in their composition of the splicing isoforms. A contribution of the e22 in the fast-twitch muscle can be proposed, whereas the Slo27 variant appears to characterize the BK channel of slow-twitch muscle (Fig. 7). It should be stressed that based on the available experimental data, the contribution of the other isoforms to the functional channel properties cannot be excluded.

In conclusion, the observed muscle phenotype-dependent BK channel biophysical and pharmacological properties correlated with variant expression levels in muscles. This finding may have relevance for conditions affecting postural muscles, such as prolonged bed-rest and microgravity conditions, or for diseases affecting fast-twitch muscles, such as periodic paralysis [45]–[46]. An altered variant ratio associated with pathological conditions may alter BK channel composition, function and drug responses.

## Materials and Methods

### Muscle Preparations and Single Fiber Isolation

Adult male Wistar rats (260–300 g) (n = 6) were used for experiments. Animal care was performed in accordance to the *Guide for the Care and Use of Laboratory Animals* by the National Academy of Sciences. The flexor digitorum brevis (FDB) and soleus (Sol) muscles were dissected from the animals under urethane anesthesia (1.2 g/kg). After dissection, the animals were rapidly killed with an overdose of urethane according to the EU Directive 2010/63/EU for animal experiments.

Single muscle fibers were prepared from FDB (n = 6) and Sol muscles (n = 6) by enzymatic dissociation in normal Ringer’s solution. After enzymatic dissociation, the fibers were used for patch-clamp experiments. The contralateral Sol (n = 6) and FDB (n = 6) muscles from the same rats were used for molecular biology experiments.

### Preparation of Total RNA and cDNA

Total RNA from rat Soleus and FDB skeletal muscles was extracted using TRIzol Reagent according to the manufacturer’s instructions (Invitrogen). RNA was treated with RNase-free DNase (Promega), and reverse transcription was performed in 17.5-microl. reactions containing 1X reverse transcriptase buffer (Invitrogen), 0.5 mM of each dNTP (Roche), 1 microM random hexamers (Amersham Biosciences), 10 units of RNasin (Promega), 10 mM DTT (Invitrogen), 4 units of Superscript II Reverse Transcriptase (Invitrogen–Life Technologies) and 1.5 microg. of total RNA. Reactions were incubated for 10 min at 25°C, 50 min at 42°C and 15 min at 70°C, and cDNA products were stored at −80°C prior to analysis.

### Transcript Scanning for Different Splice Variants

Transcript scanning was performed on total cDNA from rat tissues by PCR amplification of 7 splice sites: 6 at the C-terminus and one at the N-terminus of the Kcnma1 gene, which codes for the alpha -subunit of the BK channel. We also determined the presence of beta-subunits (Kcnmb1-4). The primers used for PCR reactions are reported in Table 2. PCR amplicons were cloned directly into the pCR2.1 TOPO vector (Invitrogen) according to the manufacturer’s instructions. Clones were digested by EcoRI, characterized by agarose gel electrophoresis and sequenced on both strands by automated sequencing (BMR Genomics, Padova, Italy).

### TaqMan™ qRT-PCR

Primers and probes for TaqMan™ quantitative real-time PCR (qRT-PCR) assays specific for each rat splice variant were designed using Primer Express version 3.0 (Applied Biosystems) using the available rat sequences for Kcnma1 (NM_031828) and beta-actin (NM_031144). Fluorescently labeled TaqMan™ probes were synthesized by Applied Biosystems. The probes were designed to amplify products encompassing each probe-annealing site as reported in Table 3. To achieve a high level of specificity and to avoid the detection of genomic DNA, we designed a probe to span exon–exon junctions for each gene. Total RNA isolated with TRIzol as described above was quantified using a spectrophotometer (NanoDrop ND-1000, Thermo Scientific). A total of 400 ng of total RNA was used for reverse transcription. cDNA synthesis was performed using random hexamers (annealed 10 min, 25°C) and Superscript II reverse transcriptase (Invitrogen–Life Technologies) at 42°C for 50 min. All assays were performed using Applied Biosystems universal cycling parameters (2 min hold at 50°C, 10 min hold at 95°C, then 40 cycles of 15 s at 95°C and 1 min at 60°C) on an Applied Biosystems ABI Prism 7300 sequence detection system. The reactions were performed in ABI Prism 96-well optical reaction plates. Each reaction contained 1X Applied Biosystems real-time PCR master mix (including ROX passive reference dye, 5 mM MgCl_2_, and nucleotides), 50 nM each of the respective forward and reverse primers and 5 nM of labeled TaqMan™ probe. Triplicate reactions were performed in parallel for each individual sample. All data were analyzed using ABI Prism 7300 SDS version 4.0 (Applied Biosystems). The results were compared with a gene-specific standard curve and normalized to the expression of the housekeeping gene beta -actin in the same sample [52]–[53]. The data for splice variants were also normalized to the expression of total BK channel molecules using a region common to all of the splice variants as a probe.

### Patch-clamp Experiments

The BK channel currents and drug actions were recorded in FDB and Sol muscle fibers during voltage steps using the excised patch-clamp technique in the presence of internal 0 M, 1.3×10^−6^ M and 10^−5^ M concentrations of free Ca^2+^ ions. The drug response was evaluated at +30 mV and −60 mV. The BK channel current was recorded at 20°C and sampled at 1 kHz (filter = 2 kHz) using an Axopatch-1D amplifier equipped with a CV-4 headstage (Axon Instruments, Foster City, CA). The voltage protocol was as follows: holding potential 0 mV, prepulse of −80 mV and test pulse from −80 mV to +80 mV in 10-mV steps. The BK channel currents were identified based on their voltage dependence and Ca^2+^ response in the excised patch [50]. The currents were leak-subtracted.

The analysis of macropatch current and of the single channel parameters (single channel current, number of channels and open probability) were performed using the pClamp 10 software package (Molecular Device, U.S.A.). The criteria for accepting the data were based on the stability of the seal, with a maximum noise level of 0.6 pA at 2 kHz. No correction for liquid junction potentials was made, which were estimated to be <1.9 mV under our experimental conditions. The pipette resistance was 2.0±0.2 MOhm (n = 250), while for single channel experiments the pipette resistance was 3.5±0.1 MOhm (n = 16). In inside-out patches, the BK currents were calculated by digital subtraction of the leak currents measured for each patch in the absence of internal Ca^2+^ ions from the maximal open channel currents recorded in the presence of internal 1.3–10×10^−6^ M concentrations of free Ca^2+^ ions. The resulting ensemble BK currents were the digital average of 16 sweeps (1 sweep/voltage membrane/patch) and 18 sweeps for each FDB and Sol muscle, respectively. The criteria for including the data in the digital average were based on the stability of the seal, with a maximum noise level of 0.6 pA at 2 kHz. No correction for liquid junction potentials was made, which were estimated to be <1.9 mV under our experimental conditions.

The applied protocol for BK channel identification and for drug response tests were as follows:

1. internal KCl with no added Ca^2+^ ions
2. internal KCl with 1.3×10^−6^ M Ca^2+^ ions
3. internal KCl with 10^−5^ M Ca^2+^ ions (control)
4. internal KCl with 10^−5^ M Ca^2+^ ions + drug (2×10^−4^ M)
5. internal KCl with no added Ca^2+^ ions (washout)

No more than 4 drugs per patch were tested. The drug solutions were applied to the patches using a fast perfusion system (AutoMate, Sci. U.S.A.). Each application of drug solution was followed by a washout period of 6 s to allow the recovery of channel currents to the control values. Due to the lack of reversibility of the QUERC action following washout, this drug was tested at the end of the drug protocol test. Seal resistance was continuously monitored during patch solution exchange.

### Recording Solutions and Drugs

The pipette (intracellular) and the bath solutions contained 150 mM KCl, 5 mM ethylene glycol bis (beta -aminoethyl ether)-*N*, *N*, *N*, *N-*tetraacetic acid (EGTA), 10 mM MOPS sodium salt, pH 7.2 with MOPS acid. CaCl_2_ was added to the bath solutions to a give a free Ca^2+^ ion concentration of 1.3×10^−6^ M and 10^−5^ M. The calculation of the free Ca^2+^ ion concentration was performed as described previously. Drugs were tested at a concentration of 2×10^−4^ M, which is the concentration required for all drugs to fully activate BK channels [54]–[55].

The compounds under investigation were the following: sulfonamides: acetazolamide (ACTZ), methazolamide (MTZ), dichlorphenamide (DCP); benzo-thiadiazine/thiazole-sulfonamides: bendroflumethiazide (BFT), hydrochlorthiazide (HCT) and ethoxzolamide (ETX); flavonoids: quercetin (QUERC) and resveratrol (RESV); and benzimidazolone (NS1619). All were purchased from Sigma Chemical Co. Stock solutions of the drugs were prepared by dissolving the drugs in dimethylsulfoxide (DMSO) to a concentration of 20×10^−3^ M. Microliter volumes of the stock solutions were then added to the bath solutions as needed. DMSO did not exceed 0.01% in the bath; at this concentration, the solvent does not affect BK channel currents.

### Data Analysis

The patches were grouped based on their sensitivity to Ca^2+^ ions and drug responses. Ca^2+^ sensitivity was evaluated by observing the changes in currents following application of increasing concentrations of free Ca^2+^ ions to the excised patches as described above. The drug response was evaluated in the presence of 10^−5^ M free Ca^2+^ ions and expressed as the percentage of the number of patches containing BK channels that responded to drugs vs. the total number of patches performed. The percentage channel activation (Effect %) observed for each drug at a fixed concentration was also evaluated as follows:

Effect %  =  ((*I BK drug* - *I BK control*/*I BK control* - *I BK max*)× 100), where Effect % is the percentage current enhancement, *I BK* max is the maximal BK current recorded at +80 mV (Vm) in the absence of drugs, *I BK control* is the BK current recorded in the absence of drugs at −60 mV or +30 mV (Vm) and *I BK drug* is the BK current recorded in the presence of drug at −60 mV or +30 mV (Vm).

The percentage channel activation (Effect %) observed with increasing concentrations of Ca^2+^ ions was evaluated as follows: Effect %  =  ((*I BK high Ca^2+^- I BK Low Ca^2+/^I BK Low Ca^2+^* - *I BK max*) × 100), where Effect % is the percentage current enhancement, *I BK max* is the maximal BK current recorded at +80 mV (Vm) in the presence of 10^−5^ M free Ca^2+^ ions, *I BK Low Ca^2+^* is the BK current recorded in the presence of the lower free Ca^2+^ ion concentration at −60 mV or +30 mV (Vm) and *I BK high Ca^2+^* is the current recorded in the presence of the higher Ca^2+^ ion concentration at −60 mV or +30 mV (Vm).

BK currents were normalized to the current recorded at +80 mV (Vm) in the presence of internal 10^−5^M Ca^2+^ ions; indeed the current recorded in this experimental condition is the maximal BK channel current technically detectable in native fibers. Other conditions such as higher calcium concentration or higher membrane depolarization cause seal instability and patch rupture.

Data analysis and plots were also performed using SigmaPlot software (Systat Software, Inc., San Jose, CA). The data were expressed as mean ± S.E. unless otherwise specified. The Student’s t-test was used to evaluate differences between means at p<0.05.
